# A Critical Review on Electric Field-Assisted Membrane Processes: Implications for Fouling Control, Water Recovery, and Future Prospects

**DOI:** 10.3390/membranes11110820

**Published:** 2021-10-27

**Authors:** Yuxiang Shen, Appala Raju Badireddy

**Affiliations:** Department of Civil and Environmental Engineering, University of Vermont, Burlington, VT 05405, USA; Yuxiang.Shen@uvm.edu

**Keywords:** membrane separation processes, electrofiltration, antifouling, physical cleaning methods, energy costs analysis

## Abstract

Electrofiltration, an electric field-assisted membrane process, has been a research topic of growing popularity due to its ability to improve membrane performance by providing in situ antifouling conditions in a membrane system. The number of reports on electrofiltration have increased exponentially over the past two decades. These reports explored many innovations, such as novel configurations of an electric field, engineered membrane materials, and interesting designs of foulant compositions and membrane modules. Recent electrofiltration literature focused mainly on compiling results without a comprehensive comparative analysis across different works. The main objective of this critical review is to, first, organize, compare and contrast the results across various electrofiltration studies; second, discuss various types of mechanisms that could be incorporated into electrofiltration and their effect on membrane system performance; third, characterize electrofiltration phenomenon; fourth, interpret the effects of various operational conditions on the performance of electrofiltration; fifth, evaluate the state-of-the-art knowledge associated with modeling efforts in electrofiltration; sixth, discuss the energy costs related to the implementation of electrofiltration; and finally, identify the current knowledge gaps that hinder the transition of the lab-scale observations to industry-scale electrofiltration as well as the future prospects of electrofiltration.

## 1. Introduction

Membrane separation processes exploit a universal physical property of matter-size. Membranes are thin semipermeable layers that could filter through liquids but retain ions, molecules, and/or particles that are larger than the pore size [1]. By choosing proper operational conditions and membrane materials, the membranes could theoretically separate ions, molecules, and colloids from liquids, with sizes ranging from sub-nanometer scale to several micrometers [2]. This size selection mechanism allows membrane processes to be an attractive alternative to conventional water and wastewater treatment methods.

All membrane processes suffer from two major limitations, namely, fouling and concentration polarization that hinder wide-scale implementation of membrane technologies [3]. Fouling occurs when ions, molecules, and/or particles (*aka* foulants) deposit and accumulate on the membrane surface, which ultimately builds up to a cake or gel layer that increases the required operational pressure for membrane filtration. Fouling is also a consequence of the high local concentration of foulants in the boundary layer at the membrane surface [4,5]. Concentration polarization occurs when the foulants concentrate and create an elevated concentration gradient near the membrane surface, which eventually leads to multiple effects in membrane filtration including an exacerbated fouling issue, back diffusion towards bulk solution, and an increased osmotic pressure gradient to overcome [3,6]. Concentration polarization frequently occurs in high pressure-driven membrane processes, including nanofiltration and reverse osmosis [7,8]. Thus, the extent of fouling and/or concentration polarization is determined by complex interactions, between the foulants and the membrane material, that are governed by water chemistry, membrane types and properties, and hydrodynamic conditions.

To address the above limitations, membrane processes commonly employ physical and chemical strategies to remove the foulant layers and/or reduce concentration polarization in membrane systems [9]. In physical cleaning, fluid shear forces are used to clean membrane surface in order to remove reversible foulants [10,11]. For instance, the transmembrane pressure is reversed to flush off foulants blocking the membrane pores. Alternatively, high crossflow velocities are used to scour the cake or gel layer off the membrane surface. In membrane plants, backwashing takes about 1/30 to 1/10 of operation time and at a flux about 1:1 to 3:1 to the operational flux [9]. In chemical cleaning, commercial products (e.g., acids, bases, and surfactants) are used to remove irreversible foulants and recover membrane performance [9,12]. Acids, such as oxalic, citric, nitric, hydrochloric, phosphoric, and/or sulfuric acids, are used to remove inorganic foulants, whereas oxidants and disinfectants are used to remove organic foulants and biofoulants [9]. Despite the application of both physical and chemical cleaning strategies, membrane performance would still decline over time and eventually new membrane modules must be installed to replace the fouled ones [13]. Another costly shortcoming for both traditional cleaning strategies is that they both require the filtration process to be partially halted to perform the cleaning task, which reduces the overall productivity of the process. In addition, use of clean water and chemicals also directly adds to the overall costs of membrane operation and environmental impacts.

To provide more efficient and effective antifouling methods, researchers have turned to alternative strategies to overcome fouling and concentration polarization [14,15]. Numerous studies have focused on modifying the membrane surface properties; for instance, modifications including membrane surface coatings, nanoparticle-enabled membranes, and biomimetic surface structures have been explored [16,17]. These membrane modifications have shown some promising results in lab-scale and pilot-scale studies, but they are yet to be evaluated under real-world, full-scale conditions. Many modifications specialize in addressing certain subcategories of fouling problems, for example, a lysozyme-coated membrane is effective against biofouling, but could provide little advantage over inorganic foulants; a Zr-based nanoparticle-embedded membrane can remove fluoride by adsorption, but otherwise does not mitigate foulant layer formation; a biomimetic membrane showed enhanced protein rejection properties, but is accompanied by exacerbated scaling [18,19,20]. Also, once the surface modification is achieved and the membrane module is installed, little can be done to further adjust the membrane surface to accommodate the actual operation. Therefore, a priori knowledge of the feed water composition is required for the engineers to apply this method, and the modified membrane could only be applied to selected, highly-controlled systems.

Electrofiltration, an electric field-assisted membrane process, is emerging as an attractive alternative that uses electric fields to assist in fouling mitigation during the membrane filtration [21,22,23]. The term ‘electrofiltration’ was exclusively used in discussion of electrophoresis and electroosmosis in earlier literature [24,25]. However, more recent publications expanded the usage of electrofiltration to include electrochemical reactions [21,26]. In electrofiltration, an electric field is applied across the membrane where the field is usually perpendicular to the membrane surface [27,28]. This technique can introduce in situ electrokinetic and electrochemical effects, such as electrophoresis, electroosmosis, electrolysis, electrocoagulation, and dielectrophoresis on demand [21,29]. These techniques would alter both the transport and deposition of the foulants that influence the structure of the cake or gel formation on the membrane surface. When compared to the conventional membrane processes (no electric field), the electric field-based effects would not only improve membrane performance, but also reduce the additional chemical use to control fouling. Electrofiltration is a very promising technique for the following reasons: (1) as a non-chemical method, it does not introduce additional contaminants into the permeate stream or the retentate stream, (2) application of the electric field does not interfere with continuous membrane operation, and (3) the electric field parameters (electric field strength and frequency, continuous vs. pulsed field) can be varied depending on the feed water composition, in order to mitigate membrane fouling.

The focus of this critical review is to present the reader with state-of-the-art knowledge in experimental and modeling work, knowledge gaps, and future prospects on the topic of electrofiltration. This review begins with a discussion on various mechanisms of electrofiltration, describing the current hypotheses and theory underlying the electrofiltration phenomena. Next, it describes the established and potential characterization methods, electric field configurations, and operational conditions crucial to electrofiltration. Then, it describes the modeling efforts aimed at characterizing electrofiltration. Next, it presents the knowledge that is needed to quantify the energy costs associated with electrofiltration. Finally, it provides a discussion on future prospects and conclusions in the field of electrofiltration.

## 2. Summary of Recent Electrofiltration Studies from Year 2000–2021

**Figure 1:**
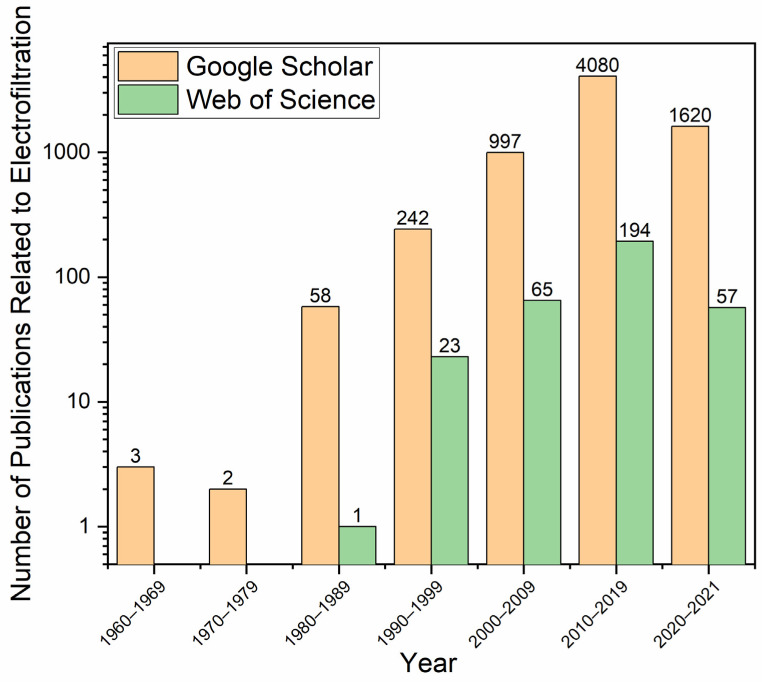
Number of results returned from Google Scholar and Web of Science by searching with ‘electric field’ and ‘membrane filtration’. Database searched included: 2; Total results shown: 7342; Numbers selected: 28.

We used Google Scholar and Web of Science with key words ‘electric field’ and ‘membrane filtration’ to extract the trend of researchers’ interest in electrofiltration and plotted the results in Figure 1. Although papers from early 2000s focused on demonstrating the proof-of-concept of electrofiltration and providing examples of exploitation of certain mechanisms to improve membrane performance, the studies in the last decade (2010s) demonstrated a growing interest to investigate novel configurations of electrodes alone or in combination with other fouling mitigation strategies, which is a *prima facie* suggestion for the feasibility of the electrofiltration as a novel fouling mitigation technique [29,30,31,32,33]. It has been shown that a low-level of electric field strength (e.g., a few millivolt/cm) is sufficient to mitigate membrane fouling, which indicates that electrofiltration, in terms of lower energy consumption, could emerge as a practical fouling mitigation strategy [31,34,35,36].

We identified several representative studies published during 2000–2021 and used them to perform the current critical review. The key highlights from the studies are summarized through Table 1, Table 2, Table 3 and Table 4. We selected these papers based on their relevance and novelty in experimental setups and analyses. Another crucial piece of information that we looked for in these papers is the exploration of the relationship between different membrane and operational parameters and flux results. During this process, we have identified numerous reports on microfiltration and ultrafiltration, which are commonly used in water and wastewater treatment and food processing industries. On the contrary, only a few studies explored the feasibility of electric fields in forward and reverse osmosis, and nanofiltration, which are commonly used in chemical processing and desalination industries. In terms of market size, microfiltration takes the largest portion of the market need (44%), followed by ultrafiltration (25%) and forward/reverse osmosis (28%), and the remaining by nanofiltration [37]. There has been a lack of standard operating procedures for researchers to follow across different studies, which makes the comparison of the results across studies difficult. Nevertheless, interesting common features, as summarized in Table 1, Table 2, Table 3 and Table 4, have been identified from different studies, including experimental parameters, filtration apparatus setups, and feed water composition and conditions. This common ground provided a basis for making comparisons among different studies. Yet, more mature methodology must be developed to account for the differences in the experiment setups, whether by analytical models to investigate the physical and chemical interactions, or by correlation analysis to statistically interpret experimental data [29,38].

## 3. Mechanisms of Electrofiltration

A lot of potential mechanisms of electrofiltration have been outlined in the literature, and they can be divided into the categories of electrokinetics and electrochemistry [59,60]. Thus far, analytical relationships have been identified to describe the electrokinetic phenomena, and empirical relationships have been applied to describe the electrochemical phenomena. The understanding of these mechanisms is crucial to the success of electrofiltration because researchers need to know the system configuration, dominant mechanisms and their effectiveness in enhancing the process. This knowledge could provide guidance for engineers to optimize the electrofiltration system performance. For the electrokinetic mechanisms, a scale analysis or an order of magnitude analysis could reduce all parameters to non-dimensional form, which would allow comparison of the relative scale of different mechanisms. For the electrochemical mechanisms, more work must be performed to develop a methodology that allows quantitative comparison to various electrokinetic processes.

### 3.1. Electrophoresis

Electrophoresis describes the phenomenon wherein charged particles or ions move along the electric field gradient under the influence of the electric field [61]. Foulants could be kept away from the membrane if the hydrodynamic force is balanced out by the electrophoretic force [62,63]. The general form describing the electrophoretic force is

(1) $$F_{E}=qE\;$$

where q is the particle charge, and E is the electric field strength [64]. The effectiveness of electrophoresis is described by electrophoretic mobility constant μ, which is defined as

(2) $$\mu =\frac{v_{e}}{E}\;$$

where $v_{e}$ is the electrophoretic velocity, and E is the electric field strength. The same term could be derived from the Helmholtz-Smoluchowski equation that

(3) $$\mu =\frac{D\varepsilon_{0}\;\zeta }{\eta }\;$$

where D is the relative permittivity or dielectric constant, $\varepsilon_{0}$ is the vacuum permittivity, ζ is the zeta potential, and η is the dynamic viscosity [26].

**Figure 2:**
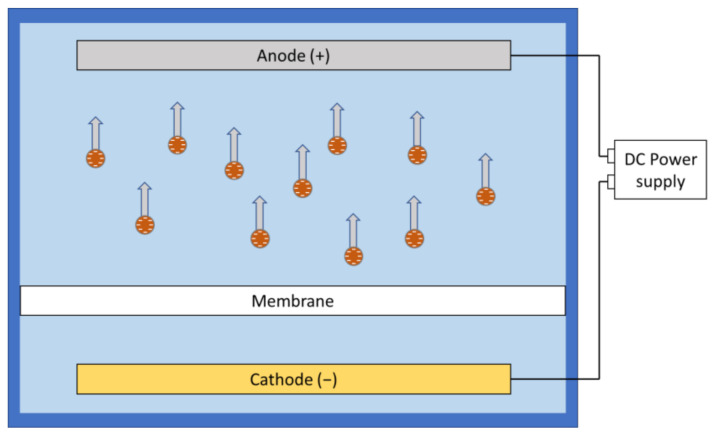
Electrophoresis of charged species under a direct current field.

Under electrophoresis, charged particles or ions will be accelerated along the electric field and potentially moved away from the membrane. In aqueous environment, particles tend to be negatively charged as the positive ions are smaller and more likely to be hydrated compared to the larger negative ions that are more likely to be adsorbed [65]. Furthermore, pH adjustment could affect the zeta potential and modify the surface charge on the foulants [28]. This enables the researchers to use simple direct current (DC) field setups to move most of the negatively charged foulants away from the membrane surface by placing the anode on the feed side of the filtration system (Figure 2). However, electrophoresis is less effective in dealing with foulants with high mass-to-charge ratio.

### 3.2. Electroosmosis

**Figure 3:**
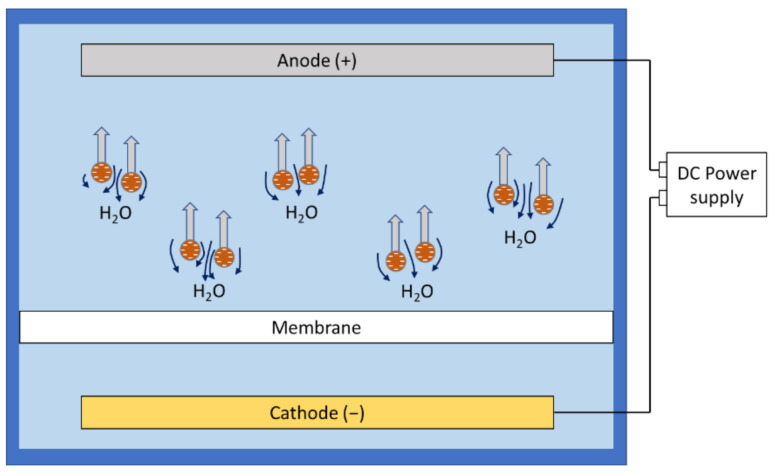
Electroosmosis under a direct current field.

Electroosmosis is the movement of a liquid relative to charged particles or membrane surface in the presence of an applied electric field [66]. (Figure 3).

Depending on the structure of the cake formation and membrane properties, electroosmosis may occur by removing the ion clouds from microchannels and inducing water displacement to fill the void [67]. It is the other commonly identified electrokinetic process in electrofiltration.

Fluid dynamics research has been performed to model the behavior of foulants under electroosmosis [68]. Kobayashi et al. studied the electroosmosis phenomenon in capillary tubes (pore diameter of 0.1 to 0.8 µm) and solved the Navier-Stokes equation, resulting in the following solution for the average liquid velocity in a vertical capillary along *z*-axis

(4) $$v_{z,av}=\frac{D\varepsilon_{0}\zeta E_{z}}{\eta }\left[\frac{2}{\alpha \left(R-\delta \right)}\frac{I_{1}\left\{\alpha \left(R-\delta \right)\right\}}{I_{0}\left\{\alpha \left(R-\delta \right)\right\}}-1\right]-\frac{\left(R^{2}-\delta^{2}\right)}{8\eta }\frac{dP}{dz}$$

where $v_{z,av}$ is the average velocity of the liquid along the *z*-axis, D is the dielectric constant of the liquid, ζ is the zeta potential as well as the potential at the slip plane, $E_{z}$ is the electric field strength along the *z*-axis, η is the viscosity of the liquid, α is the Debye–Hückel parameter, R is the capillary radius, δ is the boundary layer thickness between the capillary wall and the slip plane, P is the pressure modified by gravitational force as $\frac{dP}{dz}=\frac{dP_{L}}{dz}-\rho g$ that $P_{L}$ is the liquid pressure, ρ is the fluid density and g is the acceleration of gravity, and $I_{0}$ and $I_{1}$ are the modified Bessel function of the first kind of order zero and order one, respectively [69]. For a narrow capillary, assumption is made that the charge density along the axial direction is uniform, and this equation can be rewritten as

(5) $$v_{z,av}=\frac{\left(R^{2}-\delta^{2}\right)}{8\mu }\left(\frac{\lambda_{l}}{m_{i}}E_{z}-\frac{dP}{dz}\right)\;$$

where $\lambda_{l}$ is the specific conductance of liquid, and $m_{i}$ is the ionic mobility. When the radius of the capillary is significantly larger than the double layer (R >> δ), the equation can be simplified into

(6) $$v_{z,av}=\frac{D\varepsilon_{0}\zeta E_{z}}{\mu }-\frac{\left(R^{2}-\delta^{2}\right)}{8\mu }\frac{dP}{dz}$$

with its first term being the electrophoretic velocity by substituting in the Helmholtz-Smoluchowski equation [68]. Comparing the equation of electro-osmotic velocity of large capillary (Equation (6)) to the Helmholtz-Smoluchowski equation (Equation (3)) implies that electroosmosis is potentially at the same order as electrophoresis.

In electrofiltration, when a cake layer has already formed, it is necessary to consider electrofiltration through the porous media under the effects of electroosmosis, wherein apparent flow velocity should be used instead of flux as described by the following equation

(7) $$\frac{Q}{A}=\frac{K_{m}}{\mu }\left(\frac{I}{\phi m_{i}A}-\frac{dP}{dz}\right)=\frac{\phi^{3}}{\mu kS_{v}^{2}{\left(1-\phi \right)}^{2}}\left(\frac{I}{\phi m_{i}A}-\frac{dP}{dz}\right)$$

where Q is the flow rate, $K_{m}$ is the permeability of the media, k is the Kozeny constant, ϕ is the porosity, $S_{v}$ is the volumetric specific surface area, $m_{i}$ is the ionic mobility, and A is the cross-sectional permeation area [70].

With a clever design of experimental setups, some researchers could observe and identify electroosmosis as the most significant effect in fouling reduction. Chuang et al. used the relative size of the membrane pore and different foulant components to create a scenario where the presence of electroosmosis could be confirmed by suddenly shutting off the electric field [28]. A sudden decrease in permeate flux was observed, but the flux loss was immediately recovered with the electric field reinstalled. The application of electroosmosis in electrofiltration is highly conditional, depending on the foulant composition and membrane structure, and it may not be ready for any large-scale real-world feed water compositions.

### 3.3. Electrolysis

**Figure 4:**
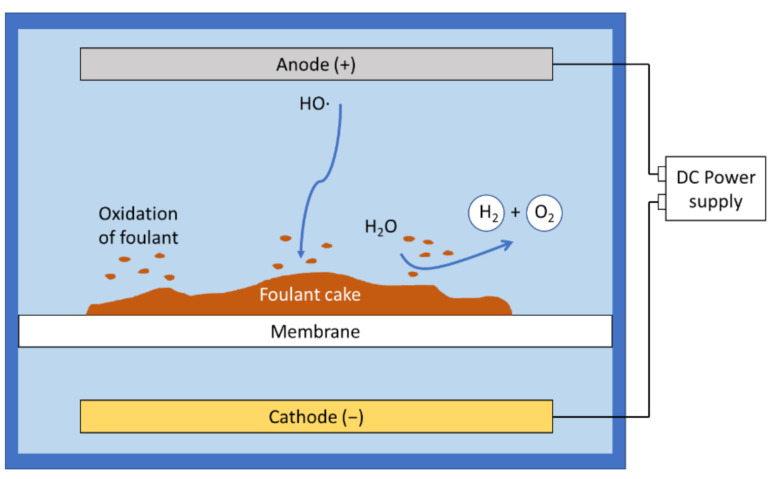
Electrolysis under a direct current field.

Electrolysis of water occurs when an electric current is passed, and gas bubbles of oxygen and hydrogen are generated due to redox reactions at the anode and the cathode (Figure 4) [44,71]. In more complex feed water compositions, other redox reactions, including generation of chlorine and/or reactive oxygen species (e.g., HO•, H_2_O_2_) are also possible [59,71,72]. The generation of gas bubbles could also mechanically break off the foulant layer due to electrolysis [73]. Liu et al. fabricated a conductive membrane embedded with reduced graphene oxide (rGO) to electrocatalytically generate hydrogen peroxide (H_2_O_2_) for removal of foulant layer from the membrane surface [32]. According to some studies, the products of electrolysis may also damage the membrane if not carefully controlled [73,74,75].

### 3.4. Electrocoagulation

**Figure 5:**
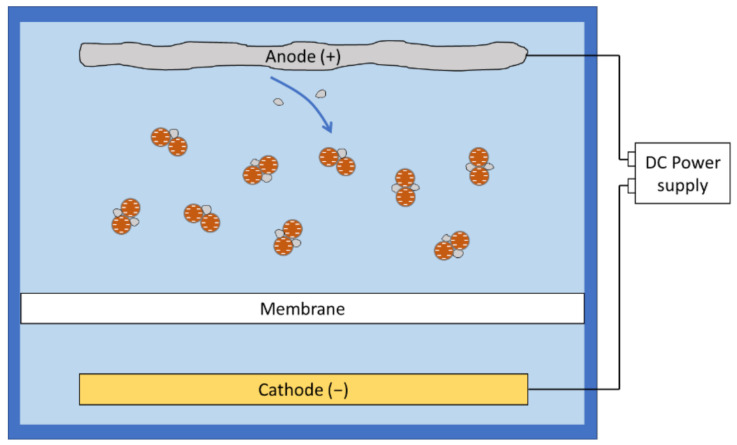
Electrocoagulation under a direct current field.

Electrocoagulation is another technique used in electrofiltration studies, wherein coagulation occurs due to the metallic ions (e.g., Fe^2+^/Fe^3+^ or Al^3+^) generated by electrochemical reactions [76]. Kobya et al. have used iron and aluminum electrodes as sacrificial electrodes to introduce Fe^2+^ or Al^3+^ based coagulants directly into the feedwater (Figure 5) [77]. Sun et al. reported a different exploitation of electrocoagulation without the application of sacrificial electrodes [53]. In the study by Sun et al., the electric field was used to enhance aggregation of natural organic matter and kaolinite particles in the feed water. In this study, while the electric field polarized the flocs and enhanced their mobility, the higher current density and lower acidic pH improved floc formation. These factors contributed to an increased porosity and polarity of the foulant cake, which in turn contributed to the increased permeate flux. For electrocoagulation, the cost of sacrificial electrodes and their replacement may be considerable, and the electrochemical reactions at electrode surfaces may hinder the performance of other mechanisms.

### 3.5. Dielectrophoresis

**Figure 6:**
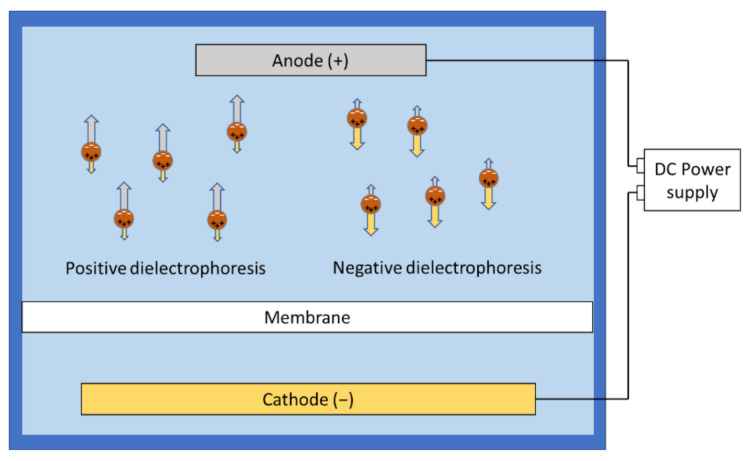
Dielectrophoresis inducing unbalanced forces on the particles/dipoles under a non-uniform direct current field.

Dielectrophoresis is another electrofiltration mechanism that is gaining attention recently. This technique exploits the non-uniform distribution of charges on foulant particles [30,42,44]. The non-uniform distribution creates a dipole moment on the particles, which can be modeled as opposite charges separated by a small distance, represented as

(8) $$\mu_{dipole}=q_{s}\cdot r_{s}\;$$

where $\mu_{dipole}$ is the dipole moment, $q_{s}$ is the separated charge, and $r_{s}$ is the separation distance between positive and negative charge. In a uniform electric field, a torque is created on the particle, because the two charges at each end experience an equal and opposite force separated by small distance. However, under a non-uniform electric field, the two charges experience forces of different magnitudes and directions, which ends up with a net force on the foulant particle (Figure 6). Molla et al. theorized using this technique to separate water droplets from water-in-oil emulsions using membranes [78]. They also tested its effectiveness in fouling mitigation for colloidal foulants [79]. The equation of dielectrophoretic force was presented as

(9) $$F_{DEP}=4\pi a^{3}\varepsilon_{0}\varepsilon_{M}re\left[K\right]\left(E\cdot \nabla \right)E$$

where $\varepsilon_{0}$ is the permittivity of free space, $\varepsilon_{M}$ is the permittivity of the medium, $re\left[K\right]$ is the real part of Clausius-Mossotti factor, a is the suspended particle radius and E is the electric field [78,79]. The Clausius-Mossotti factor, which defines the effective dielectric polarization of the foulant particle, is calculated as

(10) $$re\left[K\right]=re\left(\frac{{\bar{\varepsilon }}_{p}-{\bar{\varepsilon }}_{M}}{{\bar{\varepsilon }}_{p}+2{\bar{\varepsilon }}_{M}}\right)\;$$

(11) $$\bar{\varepsilon }=\varepsilon -\frac{i\gamma }{\omega }\;$$

where $\bar{\varepsilon }$ and ε are the complex and real permittivity, respectively; ${\bar{\varepsilon }}_{p}$ and ${\bar{\varepsilon }}_{M}$ are the complex permittivity of the particle and medium, respectively; γ is conductivity, ω is the angular frequency of the electric field, and i is the unit imaginary number. The value of $\left(E\cdot \nabla \right)E$ is approximated with $\frac{1}{2}\nabla {\left|E\right|}^{2}.$ Positive dielectrophoresis occurs if the particles have less permittivity than the medium, and negative dielectrophoresis occurs if the particles are more polarizable compared to the medium [80,81].

Researchers interested in dielectrophoresis in electrofiltration are presented with some unique opportunities as well as challenges. Unlike other mechanisms, dielectrophoresis could effectively control foulants that are not strongly charged, including neutral particles and molecules, as long as charges are not uniformly distributed on them. However, to effectively exploit dielectrophoresis, clever electrode setups are required to generate a large electric field gradient with relatively low energy, for example, carbon nanotube (CNT) based conductive membranes, or customized interdigital electrodes, which effectively reduce the separation distance between opposite electrodes [30,44]. The high electric field strength also introduces the concerns of heating, which may cause membrane damage.

### 3.6. Electrodialysis

**Figure 7:**
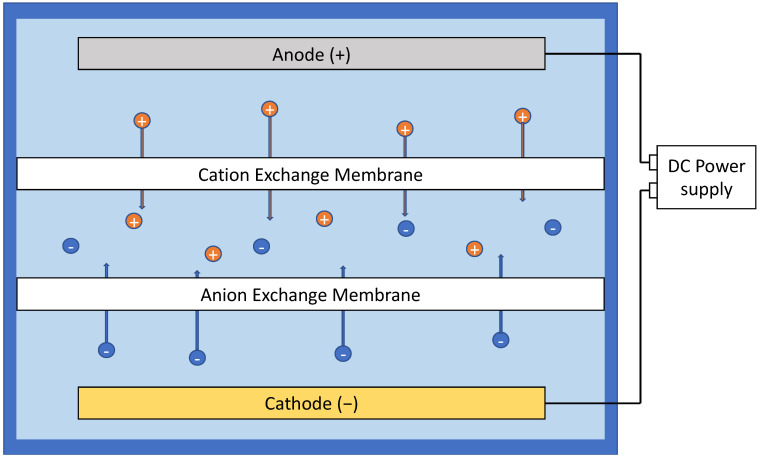
Electrodialysis under a direct current field.

Another limitedly popular concept in the literature of electrofiltration is electrodialysis. However, electrodialysis is an exploitation of electric field to aid the movement of ions in ion exchange process, involving ion-exchange membranes (Figure 7). The concept has been brought up in studies where ion exchange was coupled with ultrafiltration or reverse osmosis, but this mechanism does not directly affect the fouling behavior of membrane processes [82,83].

## 4. Characterization of Electrofiltration

The universal measurement of the performance of electrofiltration in the reported studies is the permeate flux, as it is measured by engineers in practice as an indicator of membrane performance. Permeate flux intuitively and quantitatively demonstrates the performance enhancement under different setups of electric field compared to the control groups without the field. The real-time flux measurements are obtained by recording the total weight of collected permeate at different time stages, usually collected automatically by an online system. The information obtained from flux monitoring allows researchers to develop and test various models for electrofiltration. While most researchers would only report the flux decline under continuous flow of feed water with foulants, others introduced ultrapure water again at the end of the filtration to estimate the resistance due to only the cake layer by normalizing the final resistance with the initial resistance [36,53].

Foulant rejection rate is another crucial quantitative measurement used to evaluate the membrane performance. Particulate foulants are measured as total suspended solids using a turbidity meter [31,41]. Molecular and ionic foulants and biological species are often measured using colorimetric assays and mass spectrometry [33,36,48,54,56,58].

A widely used method to qualitatively evaluate the performance of electrofiltration is visual observation of the fouling conditions on the membrane. Direct optical observation is facilitated by using transparent materials in the filtration module customization, e.g., plexiglass [35]. In addition to optical observation, scanning electron microscopy, confocal laser scanning microscopy, and other light microscopy have been applied to study and compare the membrane surface before and after fouling in ex situ analysis [31,39,44].

For researchers who fabricated membranes with specific properties, for example, electrocatalysis and conductivity, additional techniques are used to characterize the membranes. X-ray photoelectron spectroscopy is used to study the chemical functional groups on the membrane. Transmission electron microscopy is used to study the morphology of the membrane. Impedance spectroscopy is used to study the conductivity of the membrane [32,55].

Real-time monitoring of membrane fouling using quantitative tools has been gaining attention in the membrane applications [84]. In the context of electrofiltration, the application of real-time monitoring tools could provide direct information on the effects of electric field on membrane fouling. In situ visualization of fouling by magnetic resonance imaging has been reported in the more general literature for membrane filtration, however, it is yet to be applied to study electrofiltration [85,86]. Similar efforts have been reported with infrared or UV-vis spectrophotometry techniques [87,88,89,90]. Other methods with growing popularity include acoustic-based techniques [91,92,93,94]. Currently, incorporating the fouling monitoring device into the membrane system is a challenge and it may potentially interfere with the electrofiltration process. To address this challenge, a new filtration module must be designed to allow simultaneous application of the electric field and the real-time monitoring of fouling. For example, magnetic resonance imaging or ultrasound could potentially be more plausible in electrofiltration studies for real-time monitoring, but their cost and large-scale feasibility is a concern due to a combined effect of the specific requirements of membrane modules, monitoring techniques, and the data processing technologies [95].

## 5. Effect of Operational Conditions on Electrofiltration

**Figure 8:**
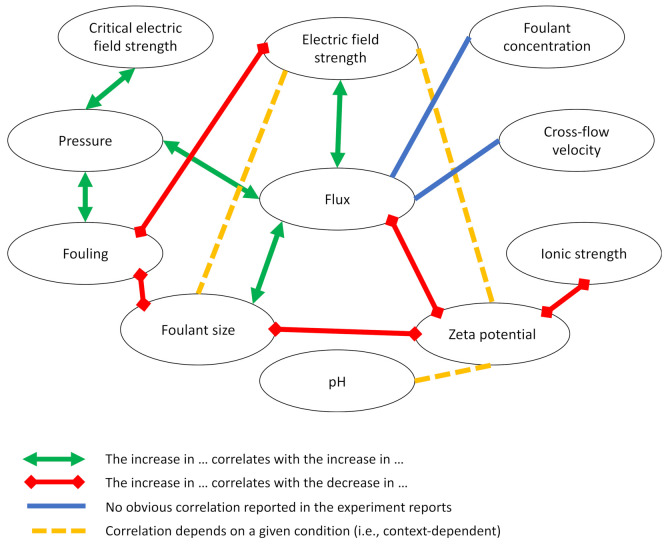
Summary of effects of various experiment factors on fouling and flux from the electrofiltration literature.

In the studies of electrofiltration, researchers have identified multiple factors that might influence the results of electrofiltration. These factors could be attributed to the design of the membrane filtration device, the installation of the electric field device, the setup of the electric field, and membrane and electrode material properties. Aspects related to the hardware devices were rarely reported as a variable in experiment designs with a few exceptions, and most of the studies altered the parameters related to electric field, membrane, and water composition to investigate the electric field effects. However, the relationships between these parameters and the results were often complicated by the fact that some parameters are not completely independent of each other in the experiments (Figure 8).

### 5.1. Configuration and Installation of Membrane Modules and Electric Fields

#### 5.1.1. Configuration of Membrane Module

Since 2000, dead-end membrane filtration studies are rare in the context of electrofiltration. Unlike the crossflow filtration where the feed flow has a component parallel to the membrane and another perpendicular to the membrane, dead-end filtration only has a feed flow component perpendicular to the membrane (therefore with smaller vortices or turbulence), which means all foulants are transported towards the membrane. Furthermore, due to lack of shear force from the crossflow, foulant accumulation is exacerbated in dead-end filtration [96,97]. Therefore, in electrofiltration experiments, dead-end filtration usually has, both spatially and temporally, a smaller scale due to rapid fouling effects. For flux monitoring in constant pressure experiments, a shorter experimental duration, which is achieved by using higher foulant concentration, may present difficulties, including but not limited to termination of the experiment before achieving critical flux, limitations on electric field setup design, and challenges of scale-up for long-term real-world operation. However, when monitoring transmembrane pressure (in constant flux experiments), a quicker buildup of transmembrane pressure occurs in dead-end filtration [44]. Also, the quicker foulant buildup allows faster production of post-filtration membrane samples for microscopy and spectroscopy analyses [32]. Flat sheet membrane setup is particularly convenient where the membrane module is customized to perform proof-of-concept data, for example, testing a novel conductive membrane or a special electrode configuration [30,54]. It also allows an easier modeling of filtration phenomenon [50]. On the contrary, hollow fiber membrane modules from manufacturers allow the researchers to investigate more realistic setups for electrofiltration [57,58].

#### 5.1.2. Installation of the Electric Field Source Ahead of the Membrane Module

Installation of the electric field ahead of the membrane module, for pretreatment of the foulants, showed benefits other than minimizing the fouling problem only. Combination of the electric field pretreatment with ultrafiltration has been reported in food processing as a method to increase the product quality [33,98,99]. These studies also reported that such an installation of placing the electric field treatment separated from and ahead of the membrane module still affects the fouling behavior. Rajha et al. reported that a pretreatment with high voltage electrical discharge or pulsed electric field has enhanced the recovery of polyphenol from vine shoot [33]. While their setup increased the product recovery rate, the additional debris from the pretreatment increased the fouling on the membrane. A study by Zhu et al. on chicory juice confirmed a similar hypothesis concerning the electric pretreatment that smaller-sized debris contributed to fouling [98]. In another study by Mhemdi et al., the researchers also reported use of pulsed-electric field to enhance sugar beet vaporization, and they also observed different fouling behaviors due to different pretreatment setups while the electric field consistently provided increased juice production [99].

#### 5.1.3. Installation of the Electric Field over the Membrane Module

Placing electrodes above and below the membrane sheet to create an electric field perpendicular to the membrane is the most commonly used setup. Some studies with hollow fiber membrane modules revealed that a simple installation of parallel electrode plates bound next to the entire module, instead of customized to fit around the hollow fibers, could provide similar fouling mitigation effects to the flat plate crossflow membrane setups [41,46]. In this configuration, the hollow fibers are submerged in the electric field, wherein, the electrophoretic forces are used to move foulant away from the membrane surface, thus leaving a cleaner surface for water permeation. The results in this study are also consistent with the electrofiltration theory that the flux enhancement is a function of electric field strength. This suggests, in principal, such a simple installation could be effective for real-world membrane applications, where hollow fiber modules are dominant [100].

#### 5.1.4. Using the Membrane as an Electrode

A customized conductive membrane as a flat sheet parallel electrode ensures the electric field is perpendicular to the membrane. This setup could be more energy saving as the separation distance between the electrodes is shortened compared to the setup with electrodes sandwiching the membrane, and it does not compromise fouling mitigation effects by removing the electric field presence on the permeate side. Another characteristic of conductive membrane is the potential to utilize electrochemical reactions at the membrane surface, which may introduce both the benefits and shortcomings of this mechanism [32,73,74]. Liu et al. reported a study of ultrafiltration with customized conductive poly(aminoanthraquinone)/reduced graphene oxide nanohybrid blended polyvinylidene difluoride (PVDF) membrane, where electrolysis of oxygen and formation of 8.84 mg/L H_2_O_2_ was observed, as well as electrostatic repulsive force was expected at the membrane [32]. Hashaikeh et al. also mentioned the air-scouring effect of gas bubbles generated in electrolysis on a multi-wall carbon nanotube coated membrane [73]. This approach depends on advances in material science for stability and cost control of conductive membrane, which needs to provide a large filtration area for real-world applications.

#### 5.1.5. Interdigital Electrodes at the Membrane

**Figure 9:**
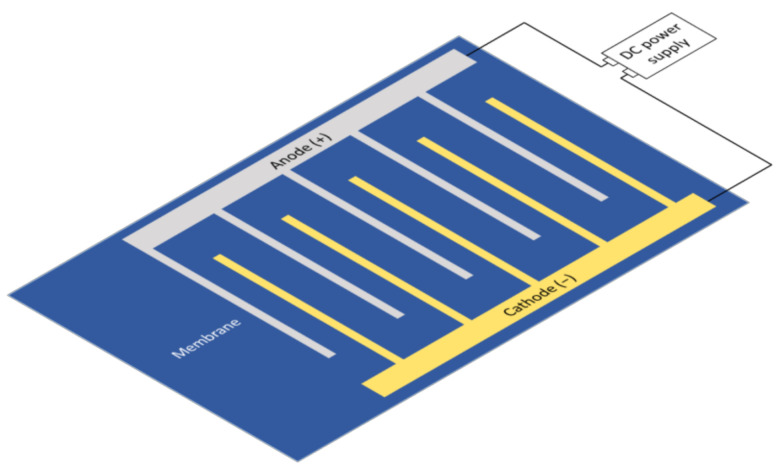
Interdigital electrodes set near the membrane surface.

A novel electrode setup has been introduced to electrofiltration by Du et al. to generate a strong electric field near the surface of the membrane with relatively small energy consumption [30]. Interdigital electrodes are composed of two-comb shaped electrodes with the teeth inserting into the empty space of the other on the same plane (Figure 9). The small separation distance (1 mm) between the teeth of opposite electrodes enabled a strong electric field strength and nonuniform field at locations that are not coplanar with the electrodes. They observed that the dielectrophoresis, although effective in moving away foulants in the bulk solution, per se, is not sufficient to remove the cake layer that has been formed on the membrane surface. A limitation of this approach is the cost of the interdigital electrodes, due to size and corrosion, respectively. However, the combination of dielectrophoresis with the conductive membrane could achieve the benefits of both setups. Zhang et al. demonstrated an example by vacuum filtration of CNT onto a PVDF membrane to demonstrate its effectiveness against biofouling [44]. Their results suggested a less orderly displacement of the interdigital electrodes was effective under the conditions of the experiments.

### 5.2. Parameters Related to the Electric Field Parameters

#### 5.2.1. Electric Field Mode

DC field is the simplest and most straight-forward setup in electrofiltration. However, it has the potential to damage the membrane due to electrolysis [73,74,75]. In membrane bioreactors, direct current has been reported to damage microbial communities as well [34]. This disadvantage led to a tendency for researchers to use low intensity DC fields [32,53]. It also invoked researcher’s interest in AC field-based electrofiltration, where the oscillation of foulants in the feed solution reduced the fouling problem [101]. However, due to the oscillatory behavior of the foulants, Zumbusch et al. also suggested AC field could not reach a higher foulant concentration in the retentate, despite mitigating the fouling behavior [101]. This suggests that in AC field setup the researchers should consider effects beyond the electrokinetics.

Another potential advantage for the application of AC field is to generate a magnetic field. The magnetic field strength can be related to the electric field by Ampere–Maxwell’s equation that

(12) $$\nabla \times H=J+\frac{\partial D_{ind}}{\partial t}$$

where H is the magnetic field strength, J is the current density, $D_{ind}$ is the electric displacement field, and t is time. There are several reports investigating the use of electromagnetic fields in membrane filtration, especially for mineral scaling control [53,57,102]. Most electrofiltration studies in the literature that we reviewed omitted discussion on the potential effects of magnetic fields because in these setups the applied current densities are not large enough to generate magnetic field strength, which could have a significant impact on foulants. The studies that explored the application of magnetic field or other magnetic mechanisms on membrane filtration deserve a separate review paper and will not be discussed here.

#### 5.2.2. Field Pulsation

Pulsed electric field was another alternative that overcomes the shortcoming of active electrochemical reactions under the DC field [103]. The pulsed electric field has also been reported as an energy saving setup compared to a continuously delivered field [31,65]. However, Hou et al. reported a study where the power input by a setup of pulsed electric field was so strong that the electrochemical reactions at electrodes hindered the performance of the membrane bioreactor [46].

From the literature, it is unclear whether the pulsed electric fields could provide better fouling mitigation effects than the continuous fields under identical experimental setups. Du et al. reported an experiment where the pulsed electric field provided better results compared to the continuous field [42]. They hypothesized that when the electric field is turned on, large particulate foulants are moved away from the fouling layer by the dominant dielectrophoretic forces. When the field is turned off, the large particles settle back onto the foulant layers and collect additional smaller foulants onto their surfaces via sorption and agglomeration. Then, when the field is turned on, foulant agglomerates are lifted and moved away by the dielectrophoretic forces. On the contrary, Chuang et al. reported that the fouling mitigation effect of the pulsed field is only as good as continuous field when the field is on. They suggested the dominant effect in their experimental setup is electroosmosis, which requires an operating electric field to be effective, rather than removal of settled foulants [28].

#### 5.2.3. Field Strength

**Figure 10:**
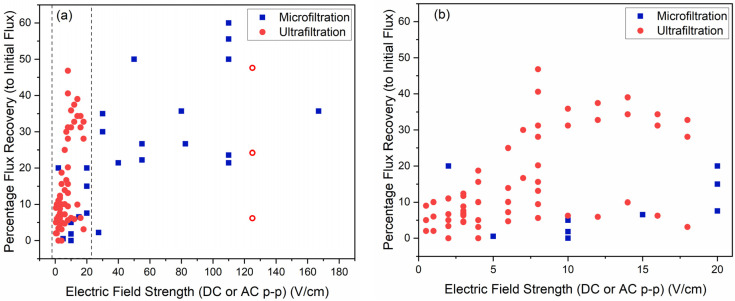
(**a**) Compilation of percentage permeate recovery vs. electric field strength. Results from DC field setups were indicated with solid symbols, and those from AC results were indicated with hollow symbols. (**b**) Magnified view of the region enclosed by the parallel dashed lines in (**a**). To date, most ultrafiltration experiments have used low-strength electric fields (<20 V/cm) and achieved higher flux recoveries. However, the electric field strength employed for microfiltration experiments were generally >20 V/cm and are more widely distributed.

Under controlled operational conditions, higher field strength results in better fouling mitigation before reaching the critical electric field strength. However, a thorough review of experimental data from several reports revealed that that the efficacy of electric field strength is highly dependent on other parameters (e.g., zeta potential, foulant size, etc.) of the electrofiltration setup (Figure 10 shows a high heteroskedasticity in the experimental data) [28,31,35,36,39,40,41,47,48,50]. The *y*-axis ‘percentage flux recovery (to initial flux)’ is calculated as

(13) $$R=\frac{J_{on}-J_{off}}{J_{o}}\times 100$$

where $J_{o}$ is the initial flux (at time, t = 0), $J_{on}$ is the reported final flux with the electric field on, under the given setup, and $J_{off}$ the reported final flux under fouling conditions (without the electric field).

Critical electric field strength is the field strength beyond which increases in fouling mitigation and transmembrane flux will not be achieved [65]. Theoretically, the critical field strength can be estimated by

(14) $$E_{crit}=\frac{J_{perm}}{\mu }$$

where $E_{crit}$ is the critical field strength, $J_{perm}$ is the permeate flux, and μ is the electrophoretic mobility of the foulant species of interest [65]. In this relation, the terminal velocity of the foulants towards the membrane is approximated with the permeate flux. Experimentally, the differences in electrophoretic mobility among foulant species is usually overlooked and an empirical value is used for the specific water composition [65,104].

The studies exploring the role of critical electric field strength on the performance of electrofiltration are limited. Earlier literature suggested the actual critical field strength is determined experimentally as the electric field strength for optimal filtration operation [65]. This is because the calculated critical electric field strength is unrealistic compared to the experimental results. Evidence suggests that the critical electric field strength is affected by various parameters in electrofiltration due to its complex interaction with the permeate flux and electrophoretic mobility. Chen et al. reported a proportional relationship between critical electric field strength and operational pressure [41]. This study suggested that the increase in operational pressure can lead to an increased sedimentation velocity, which is consistent with Sarkar and De’s study and the aforementioned proportional relationship [50]. The modeling results from Sarkar and De’s study predicted that an increase operational pressure can lead to increased critical electric field strength. On the contrary, the experimental results showed a decline in steady state flux with exceeding field strength due to the polarization effect and increased fouling, which did not agree with the results from model prediction (Equation (14)). In theory, beyond the critical electric field strength, the flux should be maintained at a constant value regardless of the field strength. Hu et al. also reported an experiment wherein, beyond certain electric field strength, the flux recovery showed a decline in electrofiltration [36]. In this study, flux decline was attributed to increased foulant aggregation and fouling due to enhanced polarization of the foulant particles in the feed solution. These results suggest that a complex interaction exists among the polarization effect of the electric field and the fouling behavior in pressure-driven membrane systems. This study also showed that critical field strength increases with increasing foulant concentration. These details are understudied in the modeling of electrofiltration phenomenon.

It is worth noting that a few electrofiltration setups were developed wherein the flux enhancement did not heavily rely on electric field strength. Sun et al.’s study on electrofiltration reported a small change (about 10%) in the initial flux in the electric field ranging from 0.5 V/cm to 2.0 V/cm [53]. They attributed this to the impacts of current density on the size, structure and polarity of foulant flocs and cake layers.

#### 5.2.4. Electric Field Gradient

Electric field gradient is reported to be an important parameter for application of dielectrophoresis in electrofiltration [30]. The magnitude of dielectrophoretic forces is proportional to the electric field gradient (Equation (9)). Dielectrophoretic systems typically use smaller separation distances between the electrodes and higher power inputs to generate a significant electric field strength over short distance.

### 5.3. Factors Related to the Filtration Setups

#### 5.3.1. Transmembrane Pressure

The effect of transmembrane pressure on the flux follows Darcy’s law

(15) $$J_{perm}=\frac{\Delta P_{TM}}{\eta (R_{m}+R_{f})}\;$$

where $J_{perm}$ is the flux, $\Delta P_{TM}$ is the transmembrane pressure, η is the dynamic viscosity, $R_{m}$ is the membrane resistance and $R_{f}$ is the fouling layer resistance [105]. To normalize the effect of transmembrane pressure, a common practice is to divide the flux by the transmembrane pressure. Darcy’s law suggests the normalized flux term is affected only by the properties of the feed water and membrane.

In electrofiltration, apart from the aforementioned effect on the critical electric field strength, another consideration regarding the transmembrane pressure appears when the foulant sizes are smaller than the membrane pore. Song et al. reported a study where 100 kDa nominal molecular weight cut-off membrane was used for 69 kDa BSA concentration [49]. Although high transmembrane pressure offered higher flux, the rejection rate decreased from 0.8 to 0.4 as the pressure increased from 0.02 MPa to 0.08 MPa. They suggested that the electrophoresis could be applied to maintain both a high flux and a high rejection rate.

#### 5.3.2. Crossflow Velocity

Crossflow setup for membrane filtration was initially developed from a dead-end setup as a method to generate a shear flow on the foulant cake or gel layer to reduce membrane fouling, and thereby obtain a better steady state flux [106]. Studies in electrofiltration confirmed this proposed effect for the crossflow component. Sarkar et al. demonstrated an experimental setup wherein the increase in crossflow velocity from 0.09 m/s to 0.12 m/s under 8 V/cm DC field increased the flux from 28.9 L/m^2^-h to 32.1 L/m^2^-h. However, a large standard deviation in results did not suggest this increase to be statistically significant [47]. The large standard deviation and, therefore, the lack of conclusive evidence is a common problem in most of the electrofiltration studies. This problem of reproducibility can be overcome by increasing the number of replicates and standardizing the protocols.

#### 5.3.3. Membrane Materials and Modifications

The fouling behavior is also a function of membrane composition and surface chemistry [107,108]. Numerous studies in electrofiltration have introduced novel membrane materials in experimental setups and demonstrated improved performance with the assistance of an electric field.

A popular category of membrane modification is the fabrication of conductive membranes or membrane coating with conductive materials [31,44,55,74,109]. Such membranes enable effective exploitation of electrolysis and electrophoresis. They also provide a potential solution for delivering the electric field in real-world large-scale applications of electrofiltration, wherein the membrane modules are much larger than the lab-scale units.

Another trend in electrofiltration comes from studies of surface modification, involving antibacterial coatings, surface microstructures, enhancement of hydrophilicity, and electro-responsiveness [110,111,112,113,114,115]. Maharubin et al. showed synergistic effects from the combination of electric field and Ag-coated membranes wherein steady state flux recovery is higher than the numerical sum of the flux recovery from each antifouling strategy alone [110]. While membrane modification provides opportunities for implementing effective antifouling effects in electrofiltration, more studies are necessary to understand the long-term effects of the electric field on the modified membrane, and how it contributes to the problem of leakage of metals or organics into the environment [116].

#### 5.3.4. Temperature

Temperature has been known to influence the membrane flux due to effects on feed fluid viscosity, and it is necessary to normalize this effect [117]. The viscosity of water at different temperature can be calculated using empirical equation between 0 °C and 35 °C

(16) $$\eta =1.777-0.052T+6.25\times 10^{-4}T^{2}\;$$

where η is water dynamic viscosity in mPa∙s, and T in °C [118]. Referring to the equations of electrophoresis (Equation (3)), electroosmosis (Equation (4)), and membrane flux (Equation (15)), the change in viscosity will affect the electrofiltration process through multiple pathways.

While most electrofiltration studies were set up under constant temperature, the low electric field strength did not induce any significant changes in temperature. There are still knowledge gaps to be closed on the effect of temperature on electrofiltration process studies.

### 5.4. Parameters Related to Water Matrix

#### 5.4.1. Zeta Potential, pH and Ionic Strength

The influence of zeta potential on membrane fouling is the effect of foulant stability. Large negative (>−30 mV) or large positive (>+30 mV) zeta potential indicates a stable aqueous suspension where flocs are less likely to form. Therefore, for unstable systems (between −30 to +30 mV), the foulants grow into large aggregates as zeta potential approaches 0 mV, which may in turn contribute to reduced membrane fouling. Statistically significant effects of zeta potential on fouling were reported by Meng et al. [38]. In a submerged electro-bioreactor study, Bani–Melhem and Elektorowicz reported a transition from stable state (−30.5 mV on average) to unstable state (up to −15.3 mV) for the feed water under the influence of an electric field [34]. The foulant modification in another potential mechanism induced in the presence of an electric field, which is an understudied phenomenon.

The pH of the feedwater directly affects the zeta potential of both the foulant particles and membrane and the interactions between them [43]. In Chiu’s 2013 study, in the absence of the electric field, the water flux recovery in microfiltration reached a local minimum at a pH where the zeta potentials of the membrane and the foulant had opposite signs, causing increased fouling [43]. It is worth noting that in the same study, the flux recovery had shifted to a different pH value when the electric field was turned on. The author attributed this phenomenon to the cancellation of the electrostatic force with the electrophoretic force. This study also suggested that the rate of flux increase due to pH increase is larger in the presence of the electric field. These results provided a basis for quantitative comparison of the relative scale of electrophoretic and electrostatic forces in electrofiltration. Venkataganesh et al. reported that the effect of change in pH, and zetapotential, is irrelevant to the steady state flux once fouling sets in [51].

A few electrofiltration studies suggested that a high ionic strength not only decreases the zeta potential of the foulant particles, but also increases aggregation of foulants [55,119]. The deposition of aggregated foulants on the membrane surface likely causes a decline in permeate flux. Another disadvantage of a high ionic strength is that the high conductivity of the feed water reduces the available voltage to be applied across electrodes leading to increased energy consumption [120].

#### 5.4.2. Foulant Concentration

The relationship between foulant concentration and steady state flux in electrofiltration is more complicated than it appears. In Sarkar and De’s study, both experimental and modeling results showed that the bovine serum albumin concentration (BSA: 0.1, 1.0 and 1.5 g/L) had no effect on the steady state flux [50]. Also, the critical electric field strength appeared to be independent of concentration because the osmotic pressure did not vary significantly with foulant concentration in all tests. However, in Song et al.’s study, similar BSA concentration setups (0.5, 1.0 and 1.5 g/L) showed flux decline with increasing foulant concentration [49]. A possible reason for the contradictory results is that the former study used 30 kDa MWCO membrane for 66.5 kDa BSA, whereas the latter used 100 kDa MWCO membrane for 69 kDa BSA, wherein the pore constriction was exacerbated by the higher concentration [121]. Venkataganesh et al. showed a decline in steady state flux when the concentration of one of the foulants (surfactant in a mixture of surfactant and naphthenic acid) increased in the feed water [51]. This suggests that the concentration of the foulant per se may not affect the steady state flux, but if the constituents induce change in adhesive or cohesive interactions between foulants and the membrane, then significant variations in the steady flux could be expected.

#### 5.4.3. Foulant Size

When all other conditions are controlled, smaller foulants tend to exacerbate the fouling problem [33,48,53]. If the foulants are smaller than the pore size, then pore constriction is another possible fouling mechanism. Also, smaller foulants form less porous and, therefore, less permeable cake or gel layers.

#### 5.4.4. Foulant Materials

To date, systematic studies on the fundamental theories of interactions between foulant materials and electric field-assisted fouling mitigation in electrofiltration are lacking. Some studies demonstrated the effectiveness of electrofiltration for some specific foulant materials, for example, BSA for protein, humic acids for organic foulants, and SiO_2_ particle for inorganic foulants [31,36]. A few studies applied electrofiltration to complex water matrices to suggest its effectiveness over foulant mixtures [39,41]. However, it is worth noting that for certain water matrices, the fouling mitigation effect of electrophoresis is relatively marginal, for example, oily wastewater because of its strong fouling capacity [45].

Remarkably the foulant-foulant and foulant-membrane interactions are unique to experimental conditions, including the electric fields; therefore, the discussion in earlier paragraphs should be interpreted in the context of experiments. For instance, cathode electrodeposition paint in wastewater sticks to the membrane surface under properly applied force or voltage [52]. In this study, an increase in electric field strength or transmembrane pressure further reduced the steady state flux.

## 6. Quantification and Modeling Efforts of Electrofiltration

Modeling remains a crucial aspect to unify and compare the results from various electrofiltration studies, a need that was demonstrated in the last section. More efforts are needed to provide a quantitative tool to relate the aforementioned factors to electrofiltration results, which, in turn, provides further insights on mechanistic understanding of the process. Thus far, the progress in this area is relatively preliminary compared to the experimental studies.

### 6.1. Hermia’s Law

Hermia’s model was developed to describe pore blocking, standard blocking (deposition on pore walls), intermediate blocking, and cake formation. These fouling types are quantitatively expressed by the following law

(17) $$\frac{d^{2}t}{dV^{2}}=k{\left(\frac{dt}{dV}\right)}^{n}$$

where k and n are constants depending on the type of fouling for all above mentioned processes, t is the time of filtration and V is the volume of permeate produced [122]. Hermia’s model was originally developed for dead-end filtration, where it was assumed that the foulants in a unit volume of feed is completely separated at the membrane, and all foulants in this unit volume deposits onto the membrane. Later Hermia’s model was also applied to crossflow filtration with the same assumptions, and this analogy was confirmed by experimental data [123]. However, when electrophoretic force is introduced, the trajectories of the foulants should not be assumed to follow the flow field, i.e., the foulants within a unit mass of feed will have a different average velocity from the flow field; therefore, not all foulants will end up on the membrane [101]. This result suggests a modification to the assumptions in Hermia’s laws is needed for modeling fouling in electrofiltration.

### 6.2. Electrodynamic Modeling

**Figure 11:**
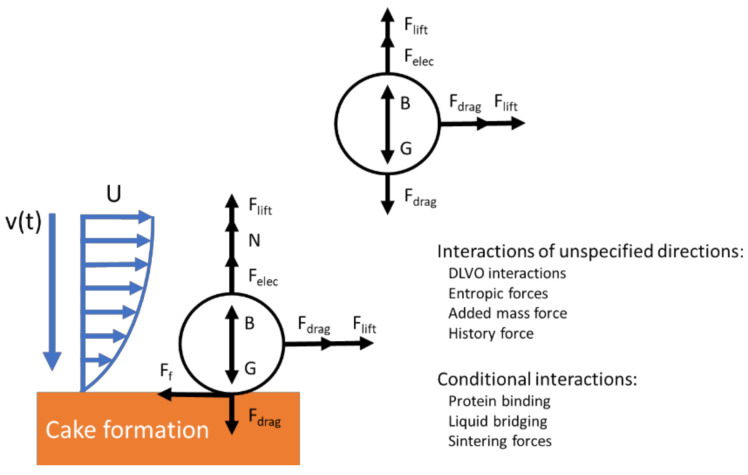
Free body diagram of foulants in electrofiltration. F_f_: friction; F_lift_: lift force; F_drag_: drag force; F_elec_: electrophoretic force; N: normal force; B: buoyancy (pressure gradient) force; G: gravitational force; v(t): flux velocity (vertical) as a function of time; U: crossflow (horizontal) profile; Derjaguin-Landau-Verwey-Overbeek (DLVO). Cake formation represents buildup of foulant layers on membrane surface.

Some researchers developed simplified free body diagrams of the interactions on a single particle to illustrate the interactions in electrofiltration [28,52,124]. These models could be effective in providing a mechanistic understanding of the foulant behavior in electrofiltration. Agana et al.’s study provided a model to describe various forces acting on a particle in the electric field. The forces acting on the foulant particle included the drag force, the lift force, the gravitational force, the buoyancy (pressure gradient) force, the van der Waals force, the electrostatic double-layer interactions, and the electrophoretic force [52]. This model assumed that electrophoresis is the dominant effect a priori and omitted other aforementioned mechanisms in electrofiltration. In some studies, the researchers have reported theoretical calculation of particle trajectory for rather specific scenarios that described their experimental setup. Molla et al. and Du et al. separately provided a trajectory calculation for particles under their dielectrophoretic setup [42,79]. For more general cases, such electrodynamic models are usually limited due to the omission of many crucial interactions, such as particle collision, forces due to unsteady flows, and Brownian motion for particulate flow in the studies of membrane filtration (Figure 11) [125]. An order of magnitude analysis may suggest some of these interactions might not be significant under certain experimental setups or real-world applications; however, such analysis has not been found in the literature in studies on electrofiltration.

In the late 20th century, the principles of fluid dynamics were applied to characterize membrane processes [126,127,128]. Recently, conventional membrane filtration studies without field assistance have also used computational fluid dynamics (CFD) to understand fouling behavior and flux recovery [129,130]. However, thus far, the development of CFD for electrofiltration is lacking, but it may gain momentum when electrofiltration is more widely used.

### 6.3. Mass Balance Modeling

A macroscale approach to model the effects of fouling mitigation is to perform a mass balance analysis on a control volume at the membrane surface. Sarkar and De provided such a model with certain assumptions and a few boundary layers [50]. They began with an equation for a 2D channel where x is the crossflow direction, and y is the flux direction:

(18) $$u\frac{\partial c}{\partial x}+\left(-v_{w}+v_{e}\right)\frac{\partial c}{\partial y}=D_{diff}\frac{\partial^{2}c}{\partial y^{2}}$$

where u is the crossflow velocity, $v_{w}$ is the flux velocity, $v_{e}$ is the electrophoretic velocity, c is the foulant concentration, and $D_{diff}$ is the diffusion coefficient. $v_{e}$ can be calculated by Helmholtz-Smoluchowski’s equation, $v_{w}$ in an osmotic pressure governed ultrafiltration is calculated as:

(19) $$v_{w}=L_{p}\left(\Delta P_{TM}-\sigma \Delta \pi \right)\;$$

where $L_{p}$ is the membrane permeability, $\Delta P_{TM}$ is the transmembrane pressure, σ is the osmotic reflection coefficient, and ∆π is the osmotic pressure across the membrane. Sarkar and De non-dimensionalized the mass balance equation and obtained analytical solutions for the terms in the mass balance equation. Starting from initially guessing the concentration at the cake layer, $c_{m}$, at a given crossflow distance x, they iteratively the calculated $c_{m}$ until the error between the guessed and calculated values converged to a minimum. The process is repeated for the overall membrane surface at a step width Δx, and a final averaged permeate flux is calculated. Their results suggested that about ±7% error between this model and measurements [50]. A shortcoming of their model is that they only considered electrophoresis as the dominant electrodynamic process, and this may limit the application of this model to certain setups and water matrices. This refers back to the earlier discussion on the lack of understanding from a perspective of fluid dynamics.

### 6.4. Simulation of Cake Layer Structure

With the advancement in computational science and simulation methods, analysis of complex process such as membrane fouling are now possible. For instance, a Monte Carlo simulation of the foulant structure has been demonstrated by Chen et al. for a simplified scenario where only mass transport, drag force, electrostatic force and van der Waal’s force were considered [131]. Based on input parameters—including Hamaker constant, surface charge, dielectric permittivity of water, permittivity of free space, temperature, and membrane resistance-steady states of foulant layer were estimated by calculating energy change in each simulation step, and the final results were visualized in terms of volume fraction. A follow-up paper by them also highlighted the limitations and knowledge gaps of their method [124]. As a deterministic model, a thorough representation of all interactions in membrane filtration is necessary for this simulation method to be accurate and useful. Efforts have been made to improve this method in latter studies by introducing fluid dynamic consideration with an updating velocity profile [132]. If researchers are interested to extend this method to study electrofiltration, more studies on the quantification of fundamental mechanisms in electrofiltration will be necessary.

Another interesting approach is to develop an empirical model based on artificial neural network (ANN) techniques for electrofiltration [133]. In an ANN process, a weight is assigned to each unit in the input layer plus a bias term in the calculation of each unit in the next hidden layer, and then the units in the hidden layer plus a bias term are calculated similarly into units in the next hidden layer or the final output layer. By providing the input and output units, the ANN model calculates the output values based on the input parameters [134]. Sarkar et al. provided an example of applying ANN to analyze the experimental results of electrofiltration [135]. The selection of parameters for the model and training—for example hidden layer number, neuron numbers, learning rate, among others—was suggested to be crucial to the ANN result. However, despite the efforts put into data cleaning and training, the obtained model may only narrowly apply to the specific experimental conditions. This limited agreement between model prediction and experimental data is likely due to the selection of input variables. For example, in a study by Sarkar et al., the researchers included feed concentration, electric field, transmembrane pressure and cross flow velocity were in the input layer, but ignored other crucial factors (e.g., membrane material, feed channel geometry, foulant components among others) [135]. This suggests a limited value in ANN for experimental design and extrapolation in electrofiltration compared to other deterministic processes.

## 7. Energy Cost Analysis

If electric fields are to be applied to control membrane fouling in the real-world setups, an important question for electrofiltration is whether it is more energy saving than conventional membrane antifouling methods. To answer this question, multiple factors need to be included into this calculation, including the direct costs to provide the hydraulic power and to generate the electric field. Additional indirect factors include opportunity costs, such as fabrication and replacement of membrane modules, fabrication and replacement of electrodes, and alternative cleaning. Thus far, studies that included all considerations to estimate the potential economic edge of electrofiltration are lacking. The challenges facing electrofiltration studies include (1) lack of standard operating procedures, and (2) lack of access to the economics knowledge behind an entire membrane operation with a complicated production and logistics chain for the science community.

However, a few studies provide insights into the cost analysis of electrofiltration, but they only focused on comparing the energy or power consumption of the hydraulic pump and the electric field generator. Unfortunately, the results are not consistent on the relative scale of these two items across the studies. In a paper by Chiu, it was reported that 0.02 kWh/m^3^ power request for pump and 1.70 kWh/m^3^ for the electric field [43]. Huotari et al. reported 110 kW/m^2^ for pump energy consumption and 0.13 kW/m^2^ for electric field [104]. Another study by Bowen et al. reported 2 kWh/m^3^ for pump operation and 0.036 to 6.9 kWh/m^3^ for the electric field operation [136]. A reasonable interpretation is that none of the comparisons is generalizable beyond the specific experimental setup. Another factor to be considered is that technology development in pumps and electric field generators have changed the power consumption of each unit.

A simple, straight-forward method was provided by Chiu to compare the energy cost of electrofiltration and traditional membrane process [43]. This method calculates the average unit energy required to produce a unit volume of permeate that

(20) $$E_{tot}=\frac{P_{p}+P_{e}}{V}\;$$

where $E_{tot}$ is the energy to produce a unit volume of permeate, $P_{p}$ is the hydraulic dissipated power, $P_{e}$ is the electrical power, and V is the volume of the permeate stream. This method allows comparison through different studies to investigate the efficiency of electrofiltration.

Therefore, it is reasonable to conclude that the energy cost analysis for electrofiltration is still in the most preliminary stages. Although examples of such analysis on certain parameters have been offered for specific bench-scale or pilot-scale setups, no efforts have been made to provide a more generalized procedure to transfer such analysis to design processes. This knowledge gap may account for the lack of full-scale electrofiltration in industrial applications. On the other hand, knowledge for maintenance and operational cost for conventional membrane processes has been well studied [12,137]. In conventional cleaning, depending on the specific scenario of water matrix and operation conditions, the cost of cleaning, including chemical cost, backwash cost, chemical heating cost, and waste treatment cost, could take as low as 2.2% of the operational cost to as high as 50.3% [138]. Electrofiltration researchers need to further optimize the system with a convincing cost analysis to push this technology to broader industrial application.

## 8. Future Prospects

The demand for clean water is expected to increase globally, and is projected to grow by a rate of 20~30% from about 4600 km^3^ to about 5500~6000 km^3^ [139]. Due to this rising demand, the use of membrane processes is also growing at a considerable rate of 8.5% in 2019, and expected to continue at a comparable rate in the following years, which will, in turn, motivate more researchers to focus on cost-effective novel fouling mitigation strategies [37,140]. Studies on electrofiltration technique have been increasing at an exponential rate in the past few decades and the results to date suggest that it could be applied to mitigate fouling in various membrane systems. Innovations in material technology, both in membrane material modification and electrode design, have been suggested to improve the performance of electrofiltration.

There are still a few factors hindering the transition of electrofiltration from lab-scale studies to real world application. Despite having a rich literature of many reports, a methodology to overcome the differences in experimental setups and compare the results is still needed, so that a design tool for electrofiltration can be developed. Previous studies have also been only providing bench-scale or pilot-scale level conclusions, and scaling up to a full-scale operation remains an under-investigated topic. A major difficulty is to effectively deliver the electric field over a large membrane module taking several square meters in wastewater treatment facilities, which requires improvement in electrode materials and electro-membrane module design. After that, a quantitative and thorough understanding of the mechanisms and design guidelines in electrofiltration needs to be developed. Last but not the least, the lack of a cradle-to-grave analysis of the cost of electrofiltration compared to traditional cleaning methods is also hindering the scale-up of electrofiltration systems. All these present many challenges to researchers interested in this topic, as well as opportunities. However, the rising need for the development of sustainable membrane processes, including non-chemical methods for fouling control, to meet future water demand and the grand challenge of providing access to clean water will make electrofiltration a very promising technology in coming decades.

## Figures and Tables

**Table 1:** Summary of electrofiltration studies using microfiltration.

Feed Water Composition	Characterization of Membrane Fouling	Electric Field	Experimental Setup	Membrane Type	Fouling Mitigation Effect	Publication Year
Prefiltered (1.2 μm) oxide-chemical-mechanical polishing wastewater: pH 9.84, conductivity 145.1 μS/cm, Total alkalinity 70 mg/L eq CaCO_3,_ TS 55.8 mg/L, Turbidity 0.39 NTU, Si 79.81 mg/L, Al 0.09 mg/L, Fe 0.12 mg/L, Cu 0.19 mg/L, Ca 0.03 mg/L, Mg 0.04 mg/L, K 21.3 mg/L	Flux monitoring; scanning electron microscopy (SEM)	Continuous direct current (DC) up to 167 V/cm; Pulsed with 10 min intervals;	Bench-scale up to 2 h; Flat plate crossflow; Parallel electrode plates (material unspecified)	Polyvinylidene difluoride (PVDF), 0.1 μm	The continuous DC field retains up to about 40% more of the initial flux by the end of the filtration; The pulsed electric field retained up to about 10% more of the initial flux by the end of the filtration.	2003 [39]
Prefiltered (0.45 μm) humic acid (aq): 1 g/L	Flux monitoring; Foulant rejection rate; SEM	Continuous DC up to 116 V/cm	Bench-scale up to 2 h; Flat plate crossflow; Parallel electrode plates (anode: platinum; cathode: titanium)	Polyethersulfone (PES), 0.1 μm	The DC field retains up to 60% of the initial flux by the end of filtration compared to without the field	2006 [40]
Activated sludge: COD 310–740 mg/L, pH 5–8, turbidity 100–500 NTU, SS 400–800 mg/L, ζ-potential −18.4~−22.6 mV, temperature 15–25 °C	Flux monitoring	Continuous DC up to 30 V/cm	Pilot-scale up to 16 h; Bioreactor with spiral hollow fiber membrane module; Parallel electrode plates (stainless steel)	Polypropylene (PP), pore size unreported	The DC field retains up to about 15% more of the initial flux by the end of filtration	2007 [41]
BSA/yeast mixture (aq): BSA 1000 ppm, yeast 1000 ppm, pH 4, 5 or 7	Flux monitoring; Foulant cake weighing; Foulant rejection rate	Continuous DC up to 50 V/cm; pulsed DC (30 s/30 s) up to 50 V/cm;	Bench-scale up to 1 h; Flat plate crossflow; Parallel electrode plates (material unspecified)	Nylon, 0.2 or 0.45 μm	The continuous DC field retains up to about 40% more of the initial flux by the end of filtration; The pulsed DC field has the similar performance compared to the continuous field when on, but slightly lower flux compared to without the field when off	2008 [28]
Clay suspension: 5 g/L, about 200 nm diameter	Flux monitoring	Continuous inhomogeneous 200 kHz alternating current peak-to-peak (AC p-p) up to 16 V/cm; Same field setup, with 10 min on and 10 min off; Same field setup, with 5 min on and 15 min off	Bench-scale up to 6 h; Flat plate crossflow; An electrode plate and a parallel grid electrode (stainless steel)	PVDF, 0.2 μm; Cellulose, 30 kDa	Compared to without an electric field, the continuous electric field took 2 times as long to reach 50% of the initial flux; The 10/10 pulsed AC field took 2.5 times as long to reach 50% of the initial flux; The 5/15 pulsed AC field took 3.3 times as long	2009 [42]
Clay suspension: 5 g/L, 100–3000 nm diameter	Flux monitoring	Continuous inhomogeneous AC field gradient up to 4.18 × 10^15^ V^2^ m^−3^	Bench-scale up to 6 h; Flat plate crossflow; Interdigitated electrodes (stainless steel)	PVDF, 0.2 μm	The continuous AC field retains up to 30% more of the initial flux compared to without the field; The pulsed AC field retains up to 50% more of the initial flux compared to without the field	2013 [30]
Whey (from bovine milk) suspension (aq): 1000 mg/L, about 1–30 μm diameter	Flux monitoring	Continuous DC field up to 20 V/cm	Bench-scale up to 60 min; Hollow fiber module crossflow; An electrode wire at the centerline of the tubular module and an electrode cylinder wrapping around the tubular module (platinum)	Ceramic, 0.2 μm	The final flux under the influence of DC field is about twice as high as without the field; The final COD in the flux under the influence of DC is about 33% more compared to that without the field	2013 [43]
Bovine serum albumin (BSA) (aq): 50 mg/L, pH 8.5; sodium alginate (aq): 50 mg/L, pH 8.5; humic acid (aq): 50 mg/L, pH 8.5; silicon dioxide particles (aq): 1000 mg/L, pH 8.5	Flux monitoring; Electrochemical impedance spectroscopy; Confocal laser scanning microscopy	2 V/cm continuous DC;	Pilot-scale up to 96 days; Bioreactor; Customized membrane with conductive mesh layer between support layer and active layer (stainless steel)	PVDF, 0.062 ± 0.024 μm	Transmembrane pressure builds up twice or thrice as fast as without the electric field; The relative flux under the electric field is enhanced to about 20% more of the initial flux.	2015 [31]
*Pseudomonas fluorescens* dispersion: 10^7^ CFU/mL	Flux monitoring; SEM	Continuous inhomogeneous DC field up to 45 V/cm; Continuous inhomogeneous 10 kHz AC field up to 45 V/cm	Bench-scale up to 1 h; Dead-end filtration; Interlaced electrodes (carbon nanotube)	PVDF, 0.3 μm	Transmembrane pressure builds up at half speed with the DC field; Transmembrane pressure builds up at one third speed with the AC field	2017 [44]
Synthetic oily wastewater	Flux monitoring; Foulant rejection rate	Continuous inhomogeneous 320 kHz AC p-p up to 270 V (field strength unspecified)	Bench-scale up to 1 h; Flat plate crossflow; An electrode plate and a parallel grid electrode (stainless steel)	Cellulose acetate, 0.45 μm	The AC field retains up to about 10% more of the initial flux by the end of filtration compared to without the field	2018 [45]
Real coal chemistry wastewater: COD 1486.4 ± 102.4 mg/L, BOD_5_ 253.3 ± 18.2 mg/L, total phenols 233.8 ± 21.2 mg/L, TOC 335.6 ± 22.3 mg/L, NH_4_-N 127.2 ± 8.5 mg/L	Foulant rejection rate; Laser diffraction particle size analyzer; Zetasizer; UV-vis spectrometer; DNA sequencing;	Pulsed direct current field current density 1.33 mA/cm^2^ with 30 s cycles, 5–10 s on	Bench-scale; Bioreactor; Parallel plate electrodes sandwiching the hollow fiber module (anode: stainless steel; cathode: graphite)	Material unspecified, 0.4 μm	With the 24 s off/6 s on field, the COD and phenol rejection rates are 83.53% and 93.28%, respectively, compared to 71.24% and 82.43% without the electric field	2019 [46]

**Table 2:** Summary of electrofiltration studies for ultrafiltration.

Feed Water Composition	Characterization of Membrane Fouling	Electric Field	Experimental Setup	Membrane Type	Fouling Mitigation Effect	Publication Year
BSA (aq): 3 or 10 g/L, 67 kDa BSA molecular weight, NaCl 0.15 mol/L	Flux monitoring	Pulsed direct current (DC) field 2 or 7 V/cm at 30 Hz	Bench-scale up to 100 min; Flat sheet crossflow; An electrode plate and a parallel grid electrode (titanium)	Polyvinylidene difluoride (PVDF), 25 kDa (~1.78 nm)	The electric field allowed about 300% increase in permeate flux	2000 [35]
Synthetic juice: pectin and sucrose 1 kg/m^3^ and 14 brix, or 3 kg/m^3^ and 12 brix, 5 kg/m^3^ and 10 brix; Natural mosambi fruit juice	Flux monitoring; Zetasizer; Spectrophotometer;	Continuous DC field strength up to 8 V/cm	Bench-scale up to 30–40 min; Flat plate crossflow; Parallel electrode plates (anode: platinum coated titanium; cathode: stainless steel)	Polysulfone (PS), 50 kDa (~2.4 nm)	The maximum electric field strength increased the final flux by ~200% compared to without the field	2008 [47]
Humic acid: 48 DOC mg/L, diameter > 3 kDa; 24 DOC mg/L, diameter 0.5 to 3 kDa; 29 DOC mg/L, <0.5 kDa	Flux monitoring; Zetasizer; UV-vis spectrometer; Atomic force microscopy (AFM)	Continuous DC up to 125 V/cm	Bench-scale up to 5 h; Flat plate crossflow; Parallel electrode plates (platinum and titanium)	Polyacrylonitrile (PAN), 100 kDa (~30 nm)	Up to 50% flux recovery for larges HA group under 125 V/cm field compared to without the electric field	2008 [48]
BSA (aq): 69 kDa, 0.5, 1 or 1.5 g/L, pH 8	Flux monitoring; UV-vis spectrometer; Zetasizer	Continuous DC up to 30 V/cm	Bench-scale up to 3 h; Flat plate crossflow; Parallel electrode plates (titanium coated ruthenium)	PS, 50 or 100 kDa	Higher electric field strength led to less concentration polarization layer resistance, higher flux and higher protein rejection rate	2010 [49]
BSA (aq): (1) 0.1 kg/m^3^, (2) 1.0 kg/m^3^, or (3) 1.5 kg/m^3^, 66.5 kDa, NaCl ionic strength 1.0 mM, pH 7.4	Flux monitoring; Zetasizer; UV-vis spectrometer;	Continuous direct current up to 20 V/cm	Bench-scale up to 40 min; Flat plate crossflow; An electrode plate and a parallel grid electrode (anode: platinum coated titanium; cathode: stainless steel)	Polyphenylene ethersulfone (PES) membrane, 30 kDa	In general, an increased transmembrane pressure and/or increased electric field strength enhances membrane filtration flux as well as an increased cake layer concentration; The theoretical model for flux provided good prediction for ±7% error	2011 [50]
Synthetic wastewater: glucose 310 mg/L, peptone 252 mg/L, yeast extract 300 mg/L, (NH_4_)_2_SO_4_ 200 mg/L, KH_2_PO_4_ 37 mg/L, MgSO_4_∙7H_2_O mg/L, MnSO_4_∙H_2_O 4.5 mg/L, FeCl_3_∙6H_2_O 0.4 mg/L, CaCl_2_∙2H_2_O 4 mg/L, KCl 25 mg/L, NaHCO_3_ 25 mg/L	Flux monitoring; Water quality monitoring;	Pulsed DC intensity 1 V/cm, 15 min on/45 min off	Pilot-scale up to 53 days; Hollow fiber module bioreactor; Electrodes are concentric hollow cylinders surrounding the membrane module (stainless steel)	Commercial membrane module, specifications unspecified	Under the electric field, membrane permeability was improved by 16.3% compared to that without an electric field	2011 [34]
Synthetic wastewater: Sodium dodecyl sulfate 8.1 mM (critical micelle concentration), naphthenic acid 500 mg/L, pH 3, 5, 7 or 9, NaCl 0, 0.01, 0.05 or 0.1 M	Flux monitoring; UV-vis spectrometer;	Continuous DC up to 10 V/cm	Bench-scale up to 1 h; Flat plate crossflow; An electrode plate and a parallel grid electrode (anode: platinum coated titanium; cathode: stainless steel)	PES, 10 kDa	In a 2 V/cm increment to 10 V/cm, up to 14% more initial flux was recovered; In a constant setup of 10 V/cm, 24% more of the initial flux was recovered	2012 [51]
Cathode electrodeposition paint (aq): 91–342 nm diameter, 5% *v*/*v*, conductivity 102.0 µS/cm, TDS 79.9 mg/L, turbidity 4644.3 NTU	Flux monitoring; Zetasizer;	Continuous DC up to 2.45 V/cm	Bench-scale up to 2 h; Crossflow with hollow fiber module; Ring electrodes at the inlet and outlet of the module, respectively, (stainless steel)	Ceramic, 50 nm	The filtration flux in electric field-assisted filtration is lower than that without the field	2012 [52]
BSA (aq): 0.5 g/L	Flux monitoring; Field emission scanning electron microscope (FESEM); Fourier transform infrared (FTIR); Contact angle analyzer; X-ray photoelectron spectroscopy (XPS); AFM	Hydraulic cleaning with 1 V/cm DC field for 2 h after filtration	Bench-scale up to 2 h; Dead-end; Electric field cleaning after filtration (reduced graphene oxide)	Synthesized poly(aminoanthraquinone)/reduced graphene oxide nanohybrid blended PVDF, ~10 nm	Fouling rate decreased by about 63.5% under the external field	2015 [32]
Vine shoot dispersion (aq): 9.09 pph (wt)	Flux monitoring; UV-vis spectrometer; High-performance liquid chromatography (HPLC);	Pretreatment with high voltage electric discharge of 40 kV at a duration of 10 µs at 0.5 Hz; Pretreatment with pulsed electric field up to 13.3 kV/cm at a duration of 10 µs at 0.5 Hz	Bench-scale; Dead-end; Electrodes composed of a needle and a plate (stainless steel)	PES, 50 kDa	Higher power input provided better break down of vine shoot and, therefore, better recovery of product polyphenol, but the increased cell break down led to more fouling	2015 [33]
Synthetic wastewater: Prefiltered (0.45 μm) humic acid 10–270 kDa, kaolinite 50 mg/L, 400–1200 nm diameter, DOC 5 mg/L	Flux monitoring; UV-vis spectrometry; SEM; Fourier transform infrared spectrometry; Particle size analyzer	Continuous DC intensity up to 20 A/m^2^, field intensity up to 2 V/cm	Bench-scale up to 15 min; Hollow fiber module crossflow; Parallel electrode plates (aluminum)	PVDF, 100 kDa (~30 nm)	Up to 50% more concentration reduction for humic acid in effluent under the electric field	2017 [53]
*E. coli* dispersion	Flux monitoring; UV-vis spectrometer; SEM	Continuous DC ~1.5 V/cm	Bench-scale up to 3 h; Flat plate crossflow; Parallel electrode plates (carbon nanotube)	Synthesized sodium lignosulfonate functionalized carbon nanotubes (CNT)/PES, ~40–60 nm	Under a weak electric field, antibacterial properties were found for the synthesized membrane; no antibacterial properties was observed without the electric field	2018 [54]
Prefiltered (5–10 kDa, 10–30 kDa, >30 kDa) humic acid (815 ± 12 mg/L); Synthetic water sample (aq): NOM from lake sediment, separated in to (1) humic acid, (2) fulvic acid, and (3) hydrophilic substances, each adjusted to 5 mg DOC/L	Flux monitoring; UV spectrometer; Total organic carbon analyzer; AFM; Contact angle analyzer; Gel permeation chromatography; SEM; FTIR	Continuous DC up to 4 V/cm	Bench-scale up to 30 min; Dead-end; Parallel electrode plates (stainless steel)	PVDF, 10 kDa (~1.42 nm)	The electric field retains up to 10% more of the initial flux compared to without the electric field	2019 [36]
Humic acid (aq): 200 ppm (wt); Humic acid w/Na_2_SO_4_: humic acid, 200 ppm (wt), Na_2_SO_4_ 0.05 M	Flux monitoring; Foulant rejection rate; Transmission electron microscopy; Linear sweep voltammetry	Continuous DC up to −0.5 V/cm	Bench-scale up to 140 min; Flat plate crossflow; Three-electrode system, the membrane as the working electrode and a parallel counter electrode (carbon nanotube)	Synthesized CNT/Al nanoparticles, 472 kDa dextran rejection (~26.9 nm)	Up to about 10% more of the initial flux retained by the electric field for humic acid solution; Up to about 5% more of the initial flux retained by the electric field for humic acid/Na_2_SO_4_ solution	2019 [55]

**Table 3:** Summary of electrofiltration studies for nanofiltration.

Feed Water Composition	Characterization of Membrane Fouling	Electric Field	Experimental Setup	Membrane Type	Fouling Mitigation Effect	Publication Year
Ibuprofen solution: 1, 10 or 20 mg/L, pH 2–7.3	UV-vis spectrometer; SEM;	Continuous direct current of 1, 2 or 3 V (field strength unspecified)	Bench-scale up to 135 min; Dead-end; Membrane as the anode and a titanium ring separated by a rubber ring as cathode	Synthesized pristine multiwalled carbon nanotubes (MWCNT) or carboxylated multiwalled carbon nanotubes (MWCNT-COOH)	Near 100% removal of ibuprofen at pH at 3 V for MWCNT-COOH, compared to 0% removal at 0 V for both membranes at pH 2 or 6	2016 [56]

**Table 4:** Summary of electrofiltration studies for forward osmosis and reverse osmosis.

Feed Water Composition	Characterization of Membrane Fouling	Electric Field	Experimental Setup	Membrane Type	Fouling Mitigation Effect	Publication Year
CaCO_3_ (aq): 5.5 mmol, pH 2–11	Flux monitoring; Salt rejection rate measuring; SEM	Continuous alternating current 25 A, 50 Hz	Pilot-scale up to 38 h; Commercial RO module; Electric circuit coils around the RO module (copper)	Commercial RO module, unspecified material	The electromagnetic field retained about 20% more of the initial flow compared to without the field after operation, and rejected 20% more salt	2016 [57]
Groundwater: TDS 5670 ± 346 mg/L, pH 7.3 ± 0.1, conductivity 6300 ± 353 µS/cm, alkalinity 222 ± 20 mg/L (CaCO_3_ eq), Chloride 538.5 ± 24.1 mg/L, sulfate 2952.5 ± 234.6 mg/L, hardness 2488 ± 42 mg/L, Magnesium 486 ± 15 mg/L, potassium ±0.2 mg/L, silicon dioxide 22.5 ± 1.6 mg/L, sodium 691 ± 74 mg/L, strontium 8.2 ± 0.2 mg/L	Flux monitoring; Ion chromatography; SEM; EDS; X-ray diffraction	Continuous random electric field by HydroFLOW, 150 kHz	Pilot-scale up to 753 h; Commercial RO module; HydroFLOW (ferrites surrounded the tubing, magnetic fields along the ferrites induced by the electric field)	Commercial RO module, polyamide	The EMF significantly reduced membrane scaling and improved RO performance by 38.3% and 14.3% in terms of normalized water permeability decline rate after 150 h and 370 h operation, respectively.	2019 [58]

## Data Availability

Data is contained within the article in Figure 10 and citations in the Section 5.2.3.

## Abbreviations

AC	Alternating current
ANN	Artificial neural network
BSA	Bovine serum albumin
CFD	Computational fluid dynamics
CNT	Carbon nanotube
DC	Direct current
MWCNT	Multi-walled carbon nanotube
PAN	Polyacrylonitrile
PES	Polyethersulfone
PP	Polypropylene
PVDF	Polyvinylidene difluoride
SEM	Scanning Electron Microscope

## Nomenclature

A	Cross-sectional permeation area (m^2^)
a	Particle radius (m)
B	Buoyancy (pressure gradient) force (N)
D	Relative permittivity/Dielectric constant
$D_{diff}$	Diffusivity (m^2^/s)
$D_{ind}$	Electric displacement field/Electric induction (C/m^2^)
E	Electric field strength (V/m)
$E_{crit}$	Critical electric field strength (V/m)
$E_{tot}$	Energy to produce a unit volume of permeate (J/L)
$E_{z}$	Electric field strength along the *z*-axis (V/m)
$F_{DEP}$	Dielectrophoretic force (N)
$F_{drag}$	Drag force (N)
$F_{E}$	Electrophoretic force (N)
$F_{f}$	Friction force (N)
$F_{lift}$	Lift force (N)
G	Gravitational force (N)
g	Acceleration of gravity (m/s^2^)
H	Magnetic field strength (A/m)
J	Current density (A/m^2^)
$J_{o}$	Initial flux (m/s)
$J_{off}$	Reported final flux with electric field off (m/s)
$J_{on}$	Reported final flux with electric field on (m/s)
$J_{perm}$	Permeate flux (m/s)
K	Clausius-Mossotti factor (F/m)
$K_{m}$	Fluid permeability of the media (d)
k	Constant in Hermia’s equation
$I_{0}$	Bessel function of the first kind of order zero
$I_{1}$	Bessel function of the first kind of order one
i	Unit imaginary number
$L_{p}$	Membrane permeability (m/s/Pa)
$m_{i}$	Ionic mobility (m^2^/s/V)
N	Normal force (N)
n	Constant in Hermia’s equation
P	Pressure (Pa)
$P_{e}$	Electrical power (W)
$P_{L}$	Liquid pressure (Pa)
$P_{p}$	Hydraulic dissipated power (W)
$\Delta P_{TM}$	Transmembrane pressure (Pa)
Q	Flow rate (m^3^/s)
q	Particle charge (C)
$q_{s}$	Separated charge (C)
R	Capillary radius (m)
$R_{f}$	Fouling layer resistance
$R_{m}$	Membrane resistance (m^−1^)
$r_{s}$	Separation distance between the opposite charges (m)
$S_{v}$	Volumetric specific surface area (m^−1^)
T	Temperature ( °C)
t	Time of filtration (s)
U	Crossflow (horizontal) profile (m/s)
u	Crossflow velocity (m/s)
V	Volume of permeate (L)
$v_{e}$	Electrophoretic velocity (m/s)
$v\left(t\right)$	Flux (vertical) as a function of time (m/s)
$v_{w}$	Flux velocity (m/s)
$v_{z,av}$	Average electroosmosis velocity along the *z*-axis (m/s)
α	Debye–Hückel parameter
δ	Boundary layer thickness between the capillary wall and the slip plane (m)
ε	Permittivity (F/m)
$\varepsilon_{0}$	Vacuum permittivity (F/m) $\varepsilon_{M}$ Permittivity of the medium (F/m)
$\varepsilon_{p}$	Permittivity of the particle (F/m) $\bar{\varepsilon }$ Complex permittivity (F/m)
${\bar{\varepsilon }}_{M}$	Complex permittivity of the medium (F/m)
${\bar{\varepsilon }}_{p}$	Complex permittivity of the particle (F/m)
ζ	Zeta potential (V)
η	Dynamic viscosity (Pa∙s)
$\lambda_{l}$	Specific conductance of liquid (S/m)
∆π	Osmotic pressure (Pa)
μ $\mu_{dipole}$	Electrophoretic mobility (m^2^/V/cm) Dipole moment (C∙m)
ρ	Fluid density (kg/m^3^)
σ	Osmotic reflection coefficient
γ	Conductivity (S/m)
ϕ	Porosity
ω	Angular frequency of the electric field (rad/s)
